# P-2142. Cross-Reactivity of Fungal Enzyme Immunoassays (EIA): Implications for Clinical Diagnosis in Patients with Autoimmune Disease

**DOI:** 10.1093/ofid/ofae631.2297

**Published:** 2025-01-29

**Authors:** Arryn Craney, Kendra Carlson, Kyla Seyfried, John Witt, Andrew Hanzlicek, Nicolas Barros, Lawrence J Wheat

**Affiliations:** MiraVista Diagnostics, Orlando, Florida; MiraVista Diagnostics, Indianapolis, Indiana; MiraVista Diagnostics, Indianapolis, Indiana; MiraVista Diagnostics, Indianapolis, Indiana; MiraVista Diagnostics, Indianapolis, Indiana; Indiana University Health / MiraVista Diagnostics, Indianapolis, Indiana; MiraVista Diagnostics, Indianapolis, Indiana

## Abstract

**Background:**

The immune system exhibits remarkable discriminatory power, distinguishing between self and non-self to mount protective responses against infections. In clinical diagnostics targeting dimorphic fungi, this immunological discrimination is leveraged to detect specific antibodies indicative of disease states. However, reliance on immune modulation for diagnostic purposes presents inherent challenges due to the intricate nature of antigen-antibody interactions. This complexity is particularly pronounced in patients with a history of autoimmune disorders, where the risk of false positive results in immunoassays is heightened due to the dysregulated immune response characteristic of autoimmune conditions.

**Methods:**

We assessed the potential for cross-reactivity of serum samples from patients with autoimmune disease in dimorphic fungal Enzyme Immunoassays (EIAs) by analyzing a cohort of clinical serum samples sourced from the Florida region, which is non-endemic for dimorphic fungal diseases. Samples were commercially sourced as remnant-banked serum samples (Medix Biochemica, Florida, USA). 25 serum samples obtained from distinct patients were subjected to testing using Coccidioides, Blastomyces, and Histoplasma EIA (MiraVista Diagnostics, Indiana, USA). This ncluded 5 individuals devoid of autoimmune disorders, cohorts of 5 patients each diagnosed with Rheumatoid Arthritis and Multiple Sclerosis, and 10 patients with Systemic Lupus Erythematosus. Samples were tested in triplicate.

**Results:**

Preliminary findings from our study unveiled nuanced levels of cross-reactivity across the dimorphic fungal EIAs utilized for the three autoimmune disorders examined. Depending on the specific dimorphic fungal EIA assay and autoimmune disorder, approximately 10–30% of serum samples from patients tested intermediate or weakly positive in the respective fungal EIAs. All control patient samples tested negative.

**Conclusion:**

These preliminary findings underscore the complexity of diagnosing dimorphic fungal infections, highlighting the significance of the patient's immune status as a confounding factor. Clinical practitioners should consider the possibility of autoimmunity when encountering weakly positive or intermediate dimorphic fungal EIA results.

**Disclosures:**

Arryn Craney, PhD D(ABMM), MiraVista Diagnostics: Employee; Salary Kendra Carlson, n/a, MiraVista Diagnostics: Employee; Salary Kyla Seyfried, n/a, MiraVista Diagnostics: Employee; Salary John Witt, n/a, MiraVista Diagnostics: Employee; Salary Andrew Hanzlicek, DVM, MiraVista Diagnostics: Employee; Salary Nicolas Barros, MD, MiraVista Diagnostics: Advisor/Consultant Lawrence J. Wheat, MD, MiraVista Diagnostics: Employee; Salary|MiraVista Diagnostics: Ownership Interest

